# Biochemistry and Immune Biomarkers Indicate Interacting Effects of Pre- and Postnatal Stressors in Pigs across Sexes

**DOI:** 10.3390/ani11040987

**Published:** 2021-04-01

**Authors:** Haley E. Rymut, Laurie A. Rund, Courtni R. Bolt, María B. Villamil, Diane E. Bender, Bruce R. Southey, Rodney W. Johnson, Sandra L. Rodriguez-Zas

**Affiliations:** 1 Department of Animal Sciences, University of Illinois at Urbana-Champaign, Urbana, IL 61801, USA; hrymut2@illinois.edu (H.E.R.); larund@illinois.edu (L.A.R.); bolt2@illinois.edu (C.R.B.); southey@illinois.edu (B.R.S.); rwjohn@illinois.edu (R.W.J.); 2 Department of Crop Sciences, University of Illinois at Urbana-Champaign, Urbana, IL 61801, USA; villamil@illinois.edu; 3 Bursky Center for Human Immunology & Immunotherapy, Washington University, St. Louis, MO 63110, USA; dianebender@wustl.edu; 4 Department of Statistics, University of Illinois at Urbana-Champaign, Urbana, IL 618012, USA

**Keywords:** biomarkers, cytokines, fasting, maternal immune activation, Poly(I:C)

## Abstract

**Simple Summary:**

This study evaluated the effects of MIA elicited by porcine reproductive and respiratory syndrome virus and postnatal metabolic or immune stressors on chemical and inflammatory biomarkers in male and female pigs. Our results provide evidence that MIA interacting with postnatal stressors can have a long-lasting effect on the pig physiology, potentially affecting health, growth, and reproductive performance later in life.

**Abstract:**

The effects of maternal immune activation (MIA) elicited by a prenatal stressor and postnatal metabolic or immune stressors on chemical and inflammatory biomarkers were studied in male and female pigs. Pigs exposed to MIA elicited by porcine reproductive and respiratory syndrome virus and matching controls were assigned at two months of age to fasting stress, immune stress, or a saline group. The serum levels of over 30 chemistry and immune analytes were studied. Significantly low levels of blood urea nitrogen were detected in females exposed to MIA, while the highest creatinine levels were identified in fasting females exposed to MIA. The levels of interferon gamma and interleukin 8 were highest in pigs exposed to postnatal immune challenge. The profiles suggest that MIA may sensitize pigs to postnatal stressors for some indicators while making them more tolerant of other stressors. Effectiveness of practices to ameliorate the impact of postnatal stressors on the physiology of the pig could be enhanced by considering the prenatal stress circumstances.

## 1. Introduction

The maternal immune response to infection or other stressors can result in fetal exposure to maternal inflammatory signals and stress hormones [1,2,3]. Maternal immune activation (MIA) during gestation can alter fetal organ developmental and immune response processes leading to the disruption in peripheral immune and metabolic molecular levels [4,5]. Concerning immune profiles, significant increases in the blood concentrations of the cytokines interleukin 1 beta (IL-1β), interferon gamma (IFN-γ), and tumor necrosis factor alpha (TNF-α), and decreases in interleukin 1 alpha (IL-1α) and interleukin 2 (IL-2) were detected in 7-day-old mice born from females that presented MIA elicited by the injection of the viral mimetic polyinosinic-polycytidylic acid (Poly(I:C)) during gestation [6]. With regard to metabolic indicators, serum non-esterified fatty acids concentrations were lower, and blood urine nitrogen concentrations were higher in heifers born from cows exposed to lipopolysaccharide immune challenge during gestation relative to control cows [7].

Porcine reproductive and respiratory syndrome virus (PRRSV) and other viruses cause substantial financial and societal losses to the pork industry [8,9,10,11], and can trigger MIA [3]. Cumulative effects of MIA and other stressors have been reported in human and rodent physiology and behavior later in life [12,13,14,15]. The double-hit hypothesis proposes that exposure to a first immune challenge during embryonic and fetal development (i.e., MIA) elicits long-term neural and immune disruptions that subsequently modify the offspring’s response to other immune challenges or stressors later in life [16]. We demonstrated that the interaction between prenatal MIA and postnatal Poly(I:C) challenge had effects on multiple behaviors including, locomotor, lethargy, and pig interactions [17]. Additionally, a study of prenatal PRRSV-elicited MIA and postnatal immune challenge elicited by lipopolysaccharides (LPS) in 28 d-old pigs detected effects of the postnatal stressor on the blood levels of TNF-α, IL-1β, and IL-10 [3].

Reports on the double-hit hypothesis in rodents and one-month-old pigs offered an initial understanding of the impact of MIA and postnatal immune stress on behavior and targeted physiological indicators of young pigs. However, advanced multifactorial and multidimensional studies still are needed to evaluate the effects of MIA across sexes and postnatal stressors on biomarker indicators of the long-term effects on pig physiology and organ function.

We postulate that pigs exposed to MIA can exhibit altered sensitivity to stressors later in life. The objective of this study is to advance the understanding of the simultaneous effects of prenatal PRRSV-elicited MIA, postnatal fasting, and immune stressors and their interactions on biomarkers of physiological health and organ dysfunction in two-month-old pigs. Around this age, pigs are typically moved from nursery phase pens to growing and finishing phase pens. This transition includes changes in management, environment, and nutrition, and therefore, metabolic and immune postnatal stressors were evaluated. 

The synergistic or antagonist effects between pre- and postnatal stressors are expected to impact blood biochemical and immune parameters because metabolic and immune stressors share molecular mechanisms, and the crosstalk between the impacted systems is fluid. The immune response to fasting is associated with the effect of the metabolic stressor on nutrient allocation to the immune system, and can interfere with pig growth and reproductive maturity [18]. Likewise, the immune response to infection can lead to inflammation-associated depression-like symptoms such as compromised feed intake. Blood cytokine and chemokine panels aid in characterizing the level and balance of pro- and anti-inflammatory signals. Blood biochemistry profiles enable monitoring the levels of minerals, metabolites, and enzymatic activity associated with liver, muscle, and kidney function [19]. The findings from this study support the implementation of management practices that alleviate the effects of pre- and postnatal stresses on pig physiology, thus positively influencing pig growth and performance.

## 2. Materials and Methods

### 2.1. Animal Experiments

The Institutional Animal Care and Use Committee at the University of Illinois approved the animal experiments. Twenty healthy, PRRSV-free PIC Camborough 22 gilts (PIC, Hendersonville, TN, USA) from the University of Illinois swine herd were artificially inseminated with semen from PIC 359 boars (PIC, Hendersonville, TN, USA), across five replicates distributed approximately 2.5 months apart. At gestation day 69, the gilts were moved into individual disease-containment chambers where they had *ad libitum* access to water, receiving daily 2.3 kg of a corn-soybean meal-based gestation diet. The nutrient compositions of the diets used are listed in Supplementary Table S1. The gilts were housed in rooms at 22 °C and exposed to a 12-h light cycle with lights on at 7:00 AM [3]. 

Following protocols proven to elicit MIA [3], on gestation day, 76 half of the gilts were intranasally inoculated with live PRRSV strain P129-BV (School of Veterinary Medicine at Purdue University, West Lafayette, IN, USA) using 5 mL of 1 × 10^5^ median tissue culture infectious dose (TCID_50_) diluted in sterile Dulbecco’s modified Eagle medium (DMEM; 5 mL total volume). The remaining gilts that served as control received 5 mL of sterile DMEM intranasally [17,20]. The gilts were snared, the snout was gently elevated, and a syringe without a needle was used for the inoculations. The gilts challenged with PRRSV received the maximum allotted daily feed allowance, and feed refusal was measured. The control gilts were fed the same amount consumed by the PRRSV-challenged gilts on the previous day. The PRRSV-challenged gilts were housed separately from the control gilts. This PRRSV challenge elicits MIA that is characterized by a significant increase in gilt body temperature and decrease in feed intake up to 10 days after inoculation [3,17,20].

Farrowing was induced on gestation day 113 using 10 mg (2 mL) injection of Lutalyse (Pfizer, New York, NY, USA), with the majority of the births occurring on gestation day 114 to 115, within the typical gilt gestation length [21,22]. Piglets were vaccinated with Excede for Swine (25 mg/pig; Zoetis, Parsippany, NJ, USA) to control respiratory diseases, with iron dextran (100 mg/pig, Butler Schein Animal Health, Dublin, OH, USA) to ensure adequate iron levels, and were not tail docked or castrated. The pigs remained with the dam in the farrowing crates until weaning at 21 days of age. Weaning did not include a feeding adaptation period, and the pigs were randomly distributed by litter to be housed in groups of four to five pigs per pen thereafter. The pigs had *ad libitum* access to water, and received a diet based on corn and soybean meal to meet the nutritional needs of growing pigs. The nutrient compositions of the diets are listed in Supplementary Table S1.

On day 59, one-third of the pigs were randomly assigned to the metabolic stress group, fasted for 24 h, and were housed separately from the rest. At 60 days of age, another third of the pigs were assigned to the immune stress group and received an injection of 1.0 mg/kg of Poly(I:C) (Sigma, St. Louis, MO, USA) and the intraperitoneal route followed published protocols [6]. The remaining third of the pigs served as control and were injected intraperitoneally with a volume of sterile phosphate-buffered saline comparable to the Poly(I:C) to ensure blind treatment assignment and were ascribed to the saline baseline group. 

At 60 days of age (approximately 100 days from gestational PRRSV-challenge), Poly(I:C) and saline injections started at 7:00 AM, and body weight was recorded one hour after injection, the time point of peak response to Poly(I:C) injection [17]. The pigs were not fed on the morning of the sampling to minimize potential postprandial effect of feeding. To facilitate blood collection under the group-housing condition, the pigs were anesthetized intramuscularly using a telazol:ketamine:xylazine drug cocktail, and the blood was drawn within 10 min via cardiac puncture. This protocol follows results from a study of the effect of anesthesia on the plasma metabolites of pigs that did not detect significant effects of ketamine:xylazine on the levels of lactate, NEFA, triglycerides, cholesterol, urea, nor glucagon within the timeframe of the present study [23]. Potential anesthetic effects on additional serum indicators were deemed negligible or comparable across all pig groups and therefore, would cancel in the contrast testing. The anesthesia cocktail ratio consisted of 50 mg of tiletamine; 50 mg of zolazepam) reconstituted with 2.5 mL ketamine (100 g/L) and 2.5 mL xylazine (100 g/L; Fort Dodge Animal Health, Fort Dodge, IA, USA) at a dose of 0.03 mL/kg body weight, following protocols [3]. Subsequently, the pigs were euthanized using an injection of sodium pentobarbital (86 mg/kg body weight, Fata Plus, Vortech Pharmaceuticals, Dearborn, MI, USA).

The experimental design followed a 2 × 2 × 3 structure including the factors of sex (female and male), prenatal stress (control and PRRSV-elicited MIA), and postnatal stress (fasting, Poly(I:C)-treated, and saline-treated). Ten pigs were measured per sex-pre-postnatal group combination for a total of 120 pigs. Poly(I:C) was selected as a postnatal immune stressor because this synthetic analog of the double-stranded viral RNA binds to the Toll-like receptor 3 and effective simulates an acute phase response to viral infection. Poly (I:C) stimulates the production of pro-inflammatory cytokines in rodents [24] and has a high degree of construct validity eliciting short-term and defined immune activation. Limited studies of the effect of Poly(I:C) in pig blood indicators have been published, and the focus has been on one-month-old animals [25,26]. The dose used in this study is proven to elicit sickness behaviors (i.e., lethargy, panting) that peak within the first hour after post-injection [17]. A dose of 0.5 mg/kg elicited changes in blood biochemistry parameters and interferon-stimulated gene expression [25,26]. 

Blood samples were collected using plastic tubes (2 × 9 mL) with clot activator and single-use needles. Blood clotting occurred at room temperature, and after that the sera were isolated by centrifugation (1300× *g* at 4 °C for 15 min) and aliquoted. The serum was stored at −80 °C and maintained in a freezer until analysis. A portion of the sera was analyzed using a clinical chemistry panel, and the rest was used for the chemokine/cytokine panel analysis.

### 2.2. Biochemistry Profiling

Reports on the effect of prenatal or postnatal immune and metabolic stressors across species suggest potential changes in multiple serum parameters. Therefore, a panel of biochemistry indicators was used to obtain a comprehensive characterization of the individual and combined stresses across time. Biochemistry measurements were obtained with a Beckman Coulter (Beckman Coulter, Inc., Brea, CA, USA) AU5400 automated chemistry analyzer that uses photometric modules to record analyte abundance (Beckman Coulter, Inc., Atlanta, GA, USA). The quantification of chemistry analytes was serviced by the Veterinary Diagnostic Laboratory at the College of Veterinary Medicine (Urbana, IL, USA). 

The panel of 13 chemistry parameters provided a comprehensive characterization of physiology, organ, and system dysfunction. Profiled analytes that can detect dysfunction on skeletal or cardiac muscle included creatine phosphokinase (CPK), aspartate aminotransferase (AST), blood urea nitrogen (BUN), and creatinine [27]. Profiled analytes that provide information on hepatic dysfunction included AST and glutamate dehydrogenase (GLDH), two enzymes known as leakage enzymes [28]. Liver function was also characterized by the levels of BUN, bilirubin, glucose, alkaline phosphatase (ALP), total protein, and albumin [29]. Profiled analytes that help in the assessment of kidney function included creatinine, calcium, phosphorus, total protein, and albumin. Profiled analytes that aid in the assessment of energy balance and metabolic system disorders included glucose, triglycerides, and cholesterol. Profiled analytes that can characterize digestive function included chlorine, sodium, potassium, total protein, and bicarbonate [19]. The levels of the biochemistry parameters were measured, and cross analytes, intra-, and inter-assay coefficients of variation were lesser or equal to 5%.

### 2.3. Immunological Profiling

The levels of 13 pro- and anti-inflammatory cytokines in the blood were measured using the MILLIPLEX MAP porcine cytokine and chemokine magnetic bead multiples assay (MilliporeSigma, Burlington, MA, USA). This multiplex bead array technology allowed the simultaneous quantification within individual samples of the levels of IFN-γ, granulocyte-macrophage colony-stimulating factor (GM-CSF), interleukin-1 receptor antagonist (IL-1ra), IL-1α, IL-1β, IL-2, interleukin 4 (IL-4), interleukin 6 (IL-6), interleukin 8 (IL-8), interleukin 10 (IL-10), interleukin 12 (IL-12), interleukin 18 (IL-18), and TNF-α. Assays were run on 25 µL of plasma following manufacturers’ instructions with standards and samples in duplicate, overnight incubation, 2-h incubation, and wash steps [30]. The assays were completed at the Bursky Center for Human Immunology & Immunotherapy Programs, Washington University School of Medicine (St. Louis, MO, USA). 

The concentration of the chemokines and cytokines were calculated using the Milliplex Analyst software Version 5.1.0.0 (Darmstadt, Germany). The measurement from wells that had at least 30 beads was used. The analyte levels were estimated from the best-fit standard curve that was inferred from known reference concentrations [30]. At least one pig group had low (average < 0.5 ng/mL) levels for IL-1ra, IL-6, IL-8, and GM-CSF and were excluded from further analysis. The final concentrations (ng/mL) were estimated by interpolations from a five-parameter model [31]. 

### 2.4. Statistical Analysis

Recognizing the crosstalk among analytes within and between metabolic and immune systems, Spearman correlations between the levels of biochemistry and cytokine parameters were computed. Interpretation of correlation coefficients is as follows: |0.9| < r < |1.0| is very strong, |0.7| < r < |0.89| is strong, |0.4| < r < |0.69| is moderate, |0.1| < r < |0.39| is weak, and |0.0| < r < |0.1| is negligible (Akoglu, 2018; Schober et al., 2018). Correlation coefficients for analytes that are part: whole relationships, such as analyte ratios and differences, were not included.

The levels of the analytes and body weight were analyzed using a linear mixed-effects model. The model included the fixed effects of prenatal (MIA and Control levels), postnatal stress (fasting, Poly(I:C)-treated, and saline-treated levels), sex, interactions between the main effects, and the random effects of gilt and replicate. The models accommodated for heterogeneity of variances across stress levels. The analyte models included the pig weight at day 60 as a covariate. 

A preliminary study of the residuals indicated that the cytokine concentrations and body weight followed a Gaussian distribution, whereas a departure from Gaussian assumptions was identified for the levels of various analytes. The natural logarithm transformation of the analyte concentrations was analyzed and offered a residual distribution consistent with the assumptions of the linear models. The analyses of the mixed effect models were implemented using the MIXED procedure with the Kenward-Rogers adjustment of degrees of freedom (SAS/STAT software, Version 9.4, 2019, SAS Institute, Cary, NC, USA). The analytical partitioning of the effect of each factor within the levels of the remaining factors was implemented using the F-test in the SLICE option within the MIXED procedure. The least square means (and standard errors) of the pre-, postnatal stress and sex groups are reported on the observed scale for body weight and cytokine concentrations, and the log-transformed scale for the biochemistry concentrations because the estimates and confidence intervals in this scale follow a Gaussian distribution. In consideration of the multiple chemical analytes studied, a False Discovery Rate (FDR) adjustment of the *p*-values was considered. In this study, a FDR-adjusted *p*-value < 0.05 corresponded to an unadjusted *p*-value < 0.01, and therefore, this threshold was considered to identify significant effects.

## 3. Results

The chemistry and cytokine concentration averages across all pig groups studied and among the saline-treated pigs from control gilts (baseline reference group) are listed in Supplementary Table S2. The Spearman correlations between indicators across all pig groups were consistent in sign and within 0.1 units of the correlation estimated from saline-treated pigs from control gilts.

Noting correlation estimates > |0.3| and excluding part: whole relationships such as analyte ratios and differences, the most extreme values were detected for BUN, glucose, and triglycerides. The correlation between BUN and phosphorus, bilirubin, triglycerides, and cholesterol was positive (0.47 < r < 0.62), whereas the correlation with glucose, sodium, chloride, and bicarbonate was negative (−0.45 > r > −0.56). Glucose was negatively correlated with creatinine, BUN, bilirubin, phosphorus, and triglycerides (−0.50 > r > −0.66). Among the lipid-associated biomarkers, triglyceride and cholesterol concentrations were positively correlated with bilirubin, creatinine, BUN, phosphorus, and cholesterol (0.41 < r < 0.65), and negatively correlated with glucose and bicarbonate (−0.43 > r > −0.63). 

Among the enzymes profiled, GGT was positively correlated with BUN (r = 0.32) and negatively correlated with glucose (r = −0.32). CPK was positively correlated with albumin, chloride, and AST (0.31 < r < 0.39), and negatively correlated with globulin, bilirubin, and triglycerides (−0.31 > r > −0.37). Among cytokines, extreme correlations (average r = 0.89) were found among IL-1α, IL-2, IL-4, and IL-10.

The most extreme correlations between chemistry and immune analytes were detected between globulin and IL-1α and IL-4 (r = −0.48 on average), followed by the correlation between albumin:globulin ratio and the same cytokines. The same profile was observed for IL-2, IL-18, and TNF-α albeit less extreme (r = −0.35 on average). Positive correlations between BUN and IL-4 (r = 0.30) were identified. 

### 3.1. Effects of Pre- and Postnatal Stressors and Sex on Serum Chemistry Analytes

A summary of the statistical significance (*p*-values) of the pre-, postnatal, sex, and interaction effects on the concentration of the chemistry analytes and body weight is presented in Table 1. Effects at FDR-adjusted *p*-value < 0.05, that corresponds to an unadjusted *p*-value < 0.01 threshold, are highlighted.

A significant three-way interaction between pre-, postnatal, and sex effects was detected for body weight and the concentrations of BUN, calcium, cholesterol, GGT, phosphorus, sodium, and triglycerides. Borderline significant effects were detected for the concentrations of ALP total, CPK, glucose, bilirubin, and protein (Table 1). The *p*-values of the lower-order model terms provide insight into the driving effects in a significant interaction. Therefore, a breakdown of the interaction effects by pre- and postnatal stressors and sex groups are summarized in Table 2 by testing the effect of Poly(I:C) within the levels of remaining factors and in Table 3 by testing the effect of MIA within the levels of the remaining factors studied. 

The columns in Table 1 correspond to the seven model terms tested. To simplify a large number of potential contrasts between the 12 pig groups studied, we present the *p*-values of biologically meaningful effects; the effect of prenatal stress within postnatal and sex pig groups (Table 2), and the effect of postnatal stress within prenatal and sex pig groups (Table 3). Most biomarkers in Table 1, Table 2 and Table 3 are annotated to shared molecular or metabolic pathways, and this is confirmed by the correlations among the parameter profiles previously enumerated. The presentation of the results from all panel indicators, at various levels of significance, highlights the complementary nature of the indicators.

Postnatal stress effects in females are driving the interaction effect on body weight (Table 2). Overall, males had the higher number of analytes significantly impacted by postnatal stress, evenly distributed across prenatal stress groups. Females exposed to a Poly(I:C) postnatal stress had the highest number of analytes significantly impacted by prenatal MIA stress (Table 3).

The effect of postnatal stress on body weight was more significant in females not exposed to prenatal stress (*p*-value < 0.019) than in females exposed to PRRSV during gestation (*p*-value < 0.029). The interaction effects for phosphorous, BUN, and calcium concentrations (*p*-value < 0.001, Table 1) were dominated by the differences in postnatal stress effects across prenatal stress-sex groups (Table 2). For example, the effect of prenatal MIA stress on phosphorous was significant among females exposed to a Poly(I:C) postnatal stress (Table 3). 

The interaction effect on triglycerides and cholesterol concentrations (*p*-value < 0.001) was extreme in males and dominated by differences in postnatal stress effects between pigs from PRRSV-challenged and control gilts. The interaction effect on GGT concentration was characterized by prenatal stress effects in Poly(I:C)-treated and fasting males (Table 2). Prenatal MIA stress had an effect on glucose concentration (Table 1), while postnatal stress had a significant effect on the concentrations of creatinine, CPK, and bilirubin (0.001 < *p*-value < 0.01).

**Figure 1:**
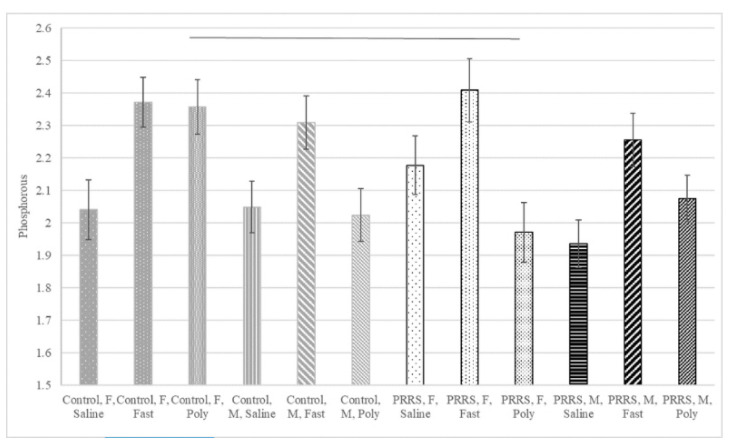
Phosphorous concentration (mg/dL, log-transformed) of 60-day-old pigs by prenatal stress (PRRSV-challenged or control gilt), postnatal stress (Poly=Poly(I:C)-treated, Saline=saline-treated, or Fast=fasting), and sex (F = female or M = male). The horizontal line depicts the significant (*p*-value < 0.05) contrast between Control and PRRSV-challenged pigs within postnatal stress and sex group (additional significant contrasts between postnatal stress and sex groups are noted in Table 4 and Table 5); whiskers denote the standard error.

**Figure 2:**
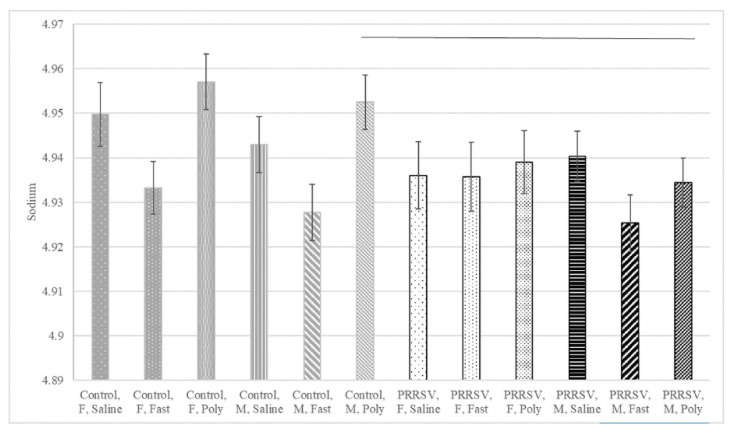
Sodium concentration (mmol/L, log-transformed) of 60-day-old pigs by prenatal stress (PRRSV-challenged or control gilt), postnatal stress (Poly=Poly(I:C)-treated, Saline=saline-treated, and Fast=fasting), and sex (F = female or M = male). The horizontal line depicts the significant (*p*-value < 0.05) contrast between Control and PRRSV-challenged pigs within postnatal stress and sex group (additional significant contrasts between postnatal stress and sex groups are noted in Table 4 and Table 5); whiskers denote the standard error.

Table 4 and Table 5 summarize the chemistry analyte concentrations and body weight by postnatal stress and sex groups in pigs not exposed (control gilts) or exposed (PRRSV-challenged gilts) to prenatal stress, respectively. The profiles of phosphorous and sodium concentrations are depicted in Figure 1 and Figure 2, respectively.

The highest variability in phosphorous and BUN concentrations were detected among pigs exposed to PRRSV prenatally, and fasting was the postnatal stressor associated with the highest concentrations of both analytes, irrespectively of sex (Figure 1). Likewise, the highest concentration of GGT was observed in PRRSV fasting males. The lowest concentrations of calcium and the highest concentrations of cholesterol and triglycerides were detected in fasting pigs, irrespective of prenatal stress or sex. 

### 3.2. Effects of Pre- and Postnatal Stressors and Sex on Serum Cytokines

The statistical significance (*p*-values) of the pre-, postnatal, sex and interaction effects on the cytokine concentrations are presented in Table 6. A significant interaction between pre-, postnatal, and sex effects was detected for IFN-γ and TNF-α (Table 6). A significant prenatal stress effect was observed for IL-18, IL-2, and IL-4, whereas a postnatal stress effect was detected for IL-12. Table 7 and Table 8 list the cytokine concentrations (estimates and standard errors) by postnatal stress and sex groups for Control and PRRSV-challenged gilts, respectively.

**Figure 3:**
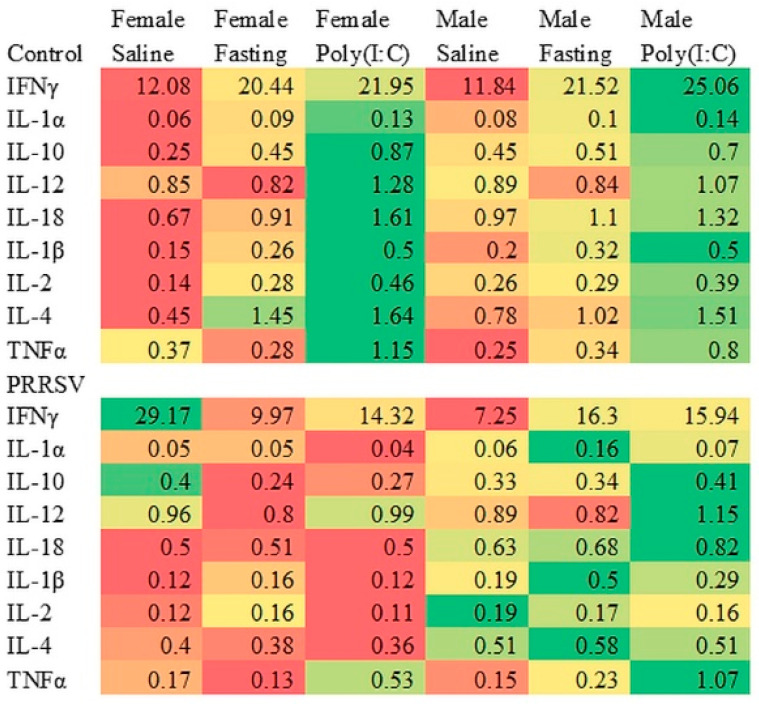
Heatmap of cytokine profiles (ng/mL) across prenatal stress (PRRSV-challenged or control gilts), postnatal stress (Poly(I:C)-treated, saline-treated, or fasting), and sex. Colors indicate a gradient of extremes to aid in visualization.IFNγ = interferon gamma; IL-1α = interleukin-1 alpha; IL-1β = interleukin 1 beta; IL-2 = interleukin 2; IL-4 = interleukin 4; IL-10 = interleukin 10; IL-12 = interleukin 12; IL-18 = interleukin 18; TNFα = tumor necrosis factor alpha. The most intense red, yellow, and green colors denote the lowest, median, and highest concentration, respectively, for each cytokine within maternal immune activation group and across the six pig groups corresponding to sex and postnatal stress combinations.

Figure 3 presents the cytokine profiles across pig groups in a heatmap characterized by the postnatal stress and sex categories for pigs from control gilts on the top and PRRSV-challenged gilts on the bottom. Heatmap cytokine values are the least square means, and red denotes low concentrations, whereas green denotes high concentrations. An overarching observation is that pigs from control gilts that were exposed to postnatal stressors at 60 days of age typically had higher cytokine concentrations (more green cells, or cells that are more intensely colored with green) relative to saline-treated pigs. Another broad observation is that Poly(I:C) had the highest impact among the stressors because of the most extreme oscillation between green and red entries detected among treated pigs. Notably, Poly(I:C)-treated females born from control gilts had the highest number of elevated cytokine concentrations characterized by the highest number of green entries. The levels of IL-2 and IL-4 were highest in Poly(I:C)-treated females from control gilts, and lowest in the counterparts from PRRSV-challenged gilts. The level of IL-4 was low (red) in pigs born from PRRSV-challenged gilts, except for fasting males. Poly(I:C) treatment was associated with high IFN-γ levels in pigs from control gilts, but this stressor did not elicit the same IFN-γ increase in pigs from PRRSV-challenged gilts.

Figure 3 also highlights that the effect of prenatal stress on the cytokine concentrations was more marked in females relative to males, as depicted by the more intense red and green hues in the former group. The effect of the prenatal stress disrupted the response of the cytokines to postnatal stress such that while the concentration of many cytokines was highest in Poly(I:C)-treated and intermediate in fasting females born from control gilts, and there was no substantial difference among females across the three postnatal stress groups (Figure 3). Postnatal stress had a significant effect on the IFN-γ concentration of males born from PRRSV-treated gilts and on the TNF-α concentration of pigs from control gilts. The highest levels of IL-10, IL-18, IL-1α, IL-2, IL-1β, and IL-12 were observed in Poly(I:C)-treated pigs, while the lowest concentrations were observed in saline-treated pigs (Table 7 and Table 8). Among the pigs from PRRSV-challenged gilts, Poly(I:C)-treated had high concentrations of six cytokines (Table 8). 

Additionally, highlighted in Table 7 and Table 8 are the differences in cytokine concentrations between sexes within pre- and postnatal groups. The concentration of IFN-γ was higher in females than males born from PRRSV-challenged gilts, and fasting males from PRRSV-challenged gilts had the highest concentrations of IL-1α.

## 4. Discussion

The present study investigated the changes in serum chemistry analyte and cytokine levels as markers of the impact of prenatal MIA stress and postnatal immune and metabolic stress. We studied both marker groups because metabolic and immune stressors share molecular mechanisms and extensive molecular crosstalk [32]. 

The average concentrations for most biochemistry analytes, including glucose, GGT, AST, creatinine, BUN, and cholesterol across all pigs and within the reference group of saline-treated pigs from control gilts, were consistent with levels reported for pigs [33]. The positive correlations between AST and GGT and between BUN and creatinine reflect that the first two enzymes are indicators of liver dysfunction [34], while the last two are indicators of renal dysfunction [35]. 

The consideration of the correlations among the biochemistry analyte profiles offered evidence of the simultaneous effect of the stressors studied on multiple physiological systems. The positive correlation between BUN and phosphorus, bilirubin, triglycerides, and cholesterol, as well as the negative correlation between BUN and glucose, calcium, sodium, chloride, and bicarbonate could reflect the mobilization of fat and protein as energy sources and generation of associated waste as a result of fasting or kidney or liver dysfunction. Many of the correlations detected in the present study were reported in other pig studies including the negative correlation between glucose and BUN [36]. The positive correlation between BUN and phosphorus, and between triglycerides and cholesterol, and the negative correlation between BUN and glucose were also reported in other species [37,38]. 

The concentrations of multiple serum cytokines, including IL-1β, IL-2, IL-4, IL-10, and TNF-α were positively correlated, consistent with the high connectivity of immune signaling pathways. In agreement with our results, TNF-α and IL-10 had consistent profiles in the hypothalamic microglia of 4-week-old pigs exposed to PRRSV during gestation and stimulated with LPS [3]. Additionally, the profiles of IL-1β and TNF-α were correlated in fetal mouse brain 4 h after MIA-elicited by Poly(I:C) and vitamin D co-administration [39]. The profiles of IL-12 and IL-18 had low correlations with the rest of the immune parameters in the present study. 

The concentrations of TNF-α, triglycerides, and total protein in saline-treated pigs from control gilts were consistent with the levels reported in the literature (Supplementary Table S2) [33,40]. The level of TNF-α in Poly(I:C)-treated pigs, and the levels of triglycerides and total protein in fasting and Poly(I:C)-treated pigs were higher than the reported average but within the ranges listed in the literature [33,40]. 

The correlations between chemistry and immune analytes across pre- and postnatal stressors confirmed the interconnection between the metabolic and inflammatory systems. The negative correlations between serum globulin and interleukin concentrations, including IL-4, is consistent with the opposite pattern between serum immunoglobulins A and M and IL-4 in weanling pigs challenged with rotavirus [41]. The magnitude of the postnatal stress effects was higher than of prenatal and sex effects. This result was anticipated because the timing of the sampling was within 24 h of the postnatal stress while 100 days from the prenatal stress. Nevertheless, the identification of many biomarkers presenting significant prenatal stress effects, alone or interacting with postnatal stress, suggests that MIA had prolonged effects that influence the response of the pigs later in life to metabolic or immune stressors. 

### 4.1. Effects of Pre- and Post-Natal Stressors and Sex on Serum Metabolic Parameters

Body weight at 60 days of age had a significant interaction between pre and postnatal stressors and sex. A marked effect of postnatal stress was detected in females, with fasting females weighing significantly less than saline- and Poly(I:C)-treated females. The most significant difference in body weight between MIA groups was detected among saline-treated pigs. In consideration that body weight was similar between MIA groups, the previous pattern suggests that prenatal stress may offer some protection from the effects of fasting stress on body weight.

Blood urea nitrogen had a significant interaction between pre- and postnatal stressors and sex. This effect was dominated by postnatal stress on males from control gilts, whereas the trend was moderate among males from PRRSV-challenged gilts. Mice exposed to renal stress saw heightened urea nitrogen changes in males relative to females [42]. Our results suggest that MIA may increase the tolerance of males to a second stressor later in life due to males being prone to high concentrations. High levels of BUN may be associated with changes in feed intake, sources of energy, and metabolism of muscle proteins, and kidney failure to remove urea [43]. Overall, the highest levels of BUN were detected in fasting pigs, and this profile is consistent with reports that higher plasma urea values were associated with low protein intake and high amino acid catabolism [44]. The lowest BUN levels were detected in Poly(I:C)-treated pigs, and the interaction effect was characterized by the lowest levels observed in males from control gilts and females from PRRSV-challenged gilts. In agreement with our results, an intravenous injection of Poly(I:C) caused an increase in BUN levels among one-month-old pigs [25,26]. 

The serum concentration of creatinine, a waste product that is generated in the muscles, had a significant postnatal stress effect. The highest levels of creatinine were detected in Poly(I:C)-treated females born from control gilts and fasting females from PRRSV-challenged gilts. Higher BUN and creatinine concentrations could indicate subclinical renal insufficiency, increased metabolic use or turnover of protein deposits as energy source. Our results are consistent with higher creatinine levels in one-month-old pigs challenged with Poly(I:C) [25,26]. 

The effect on serum glucose levels of postnatal stressors was observed in males born from control gilts, suggesting that MIA may attenuate the effect of postnatal stress on serum glucose concentrations. Fasting female and male pigs had lower levels of glucose than saline-treated pigs, and this difference reached significant levels in male pigs from control gilts. Decreased serum glucose levels in response to depressed feed consumption have been established [45]. Among the pigs born from control gilts, the level of glucose in Poly(I:C)-treated males was closer to that of saline-treated males. These trends support reports that Poly(I:C)-treated one-month-old pigs had lower levels of glucose than controls [25,26].

The concentrations of cholesterol and triglyceride presented similar sex-by-stressor interaction effects. Notable, the effect of postnatal stress on cholesterol was more marked in males from PRRSV-challenged gilts, suggesting that MIA sensitizes males to postnatal stressors that could affect lipid metabolism. Cholesterol levels were significantly higher in fasting males than in saline- and Poly(I:C)-treated males that presented similar concentrations. Consistent with these patterns, a significant increase in serum cholesterol was detected one day after initiation of fasting [46] and in pigs receiving restricted feeding [47]. The high triglyceride and cholesterol levels could be associated with a metabolic response to unmatched energy needs in fasting pigs that result in high lipid mobilization and gluconeogenesis activity. Fasting leads to negative energy balance, and triacylglycerol provides free fatty acids for oxidation that can be, in turn, converted to ketone and acetone that act as an alternative energy source to glucose [48]. Supporting our results, one-month-old pigs injected with Poly(I:C) had lower levels of cholesterol than controls [25,26]. 

The concentration of bilirubin was significantly lower in fasting males relative to other groups from PRRSV-challenged gilts. The highest bilirubin levels were detected in Poly(I:C)-treated females, and this profile agrees with the higher levels of bilirubin detected in one-month-old pigs challenged with Poly(I:C) [25,26].

The serum levels of GGT, an enzyme indicator of liver dysfunction, muscle dystrophy [44,49], and cardio complications [50], had a significant interaction between stressors and sex. The effect of a postnatal stressor was marked among males from PRRSV-challenged gilts, suggesting that MIA sensitized males to the second stress (Table 2 and Table 5). The highest GGT concentrations were detected in fasting pigs from PRRSV-challenged gilts, and this could be explained by the ability of females to pass GGT to the offspring [51]. GGT tends to increase under stress such as weaning [52]. This trend is consistent with the use of GGT as an indicator of liver and muscle damage and stress. The GGT and CPK profiles were aligned in fasting pigs. The effect of postnatal stressor on CPK was marked in males from PRRSV-challenged gilts, consistent with the lower levels of serum CPK reported in pigs fasted for 24 h [53]. 

The concentration of phosphorous had a significant interaction between pre- and postnatal stressors and sex effect. The phosphorous concentration was significantly higher in fasting relative to saline- and Poly(I:C)-treated pigs. The effect of postnatal stressors was marked in females born from PRRSV-challenged gilts, suggesting that MIA may sensitize females to postnatal stress and impact phosphorous catabolic pathways or filtering. Fasted mice had increased serum phosphorus levels [54], and elevated serum phosphorus can indicate kidney deterioration [55]. 

Among pigs exposed to MIA, higher levels of phosphorous were detected in saline-treated females than saline-treated males, whereas among pigs from control gilts, Poly(I:C)-treated females had a higher level of phosphorous than Poly(I:C)-treated males. This result suggests that immune stressors increase phosphorus levels in females irrespective of pre- or postnatal exposure. Consistent with the profiles observed in the present study, lower levels of serum phosphorus in pigs were associated with reduced feed consumption and higher excretion [45]. Increased phosphorus levels may be attributed to increased energy utilization that is observed in sick or fasting piglets to overcome the stressor [56]. Consistent with the anion gap patterns, one-month-old pigs treated with Poly(I:C) experienced an increase in phosphorous and potassium [25,26]. Kidney dysfunction and starvation can result in lower the levels of sodium, potassium, magnesium, and inorganic phosphorous.

The concentration of calcium presented a significant interaction between pre- and postnatal stress and sex. Calcium levels were impacted by postnatal stress among pigs from control gilts (Table 2 and Table 4). The weaker effect of postnatal stress among pigs from PRRSV-challenged gilts could indicate that prenatal stress enhanced the tolerance to postnatal stressors that impact calcium physiological use or removal. The effect of the stressors on calcium levels may be an indirect result of the effect on phosphorous or on lipolysis. In pigs, 10% of serum calcium is bound to non-protein anions such as phosphate and the calcium changes could be due to the higher formation of calcium and phosphate complexes [57]. Hypocalcemia was prevalent among fasting male and female pigs from control gilts and fasting pigs from PRRSV-infected gilts. Supporting these findings, decreased serum calcium concentration has been associated with reduced feed consumption and higher excretion in pigs [45]. 

Overall, the interacting and additive effect of pre- and postnatal stressors on serum biochemistry biomarkers of 60-day-old pigs were detected in the present study. For some parameters (e.g., cholesterol), MIA stress may sensitize pigs to postnatal immune or metabolic stresses. For other parameters (e.g., BUN), MIA may prime pigs to be less sensitive to postnatal stresses that affect serum markers of organ function.

### 4.2. Effects of Pre- and Postnatal Stressors and Sex on Serum Immune Parameters

The serum concentration of IFN-γ had a significant interaction between pre- and postnatal stress and sex effect. The concentration of IFN-γ was higher in Poly(I:C)-treated pigs across sexes and prenatal MIA groups. Among saline-treated pigs, the level of IFN-γ was highest in pigs experiencing MIA, and among these, females presented the highest IFN-γ concentration. This result is consistent with the higher levels of serum IFN-γ detected in one-week-old mice from dams injected with Poly(I:C) during gestation [6]. IFN-γ is secreted by activated T-cells and is essential in the activation of the innate immune system, which also explains the increase seen in pigs exposed to MIA [58]. Reports suggest that offspring exposed to MIA are born with activated inflammatory macrophages, leading to an increase in production of IFN-γ and subsequently of other cytokines [59]. Among pigs exposed to MIA, fasting, and Poly(I:C)-treated pigs had the lowest levels of IFN-γ. This trend suggests that prenatal MIA stress may lower the response of IFN-γ to postnatal stressors.

The serum levels of IL-12 and TNF-α were higher in Poly(I:C)-treated pigs irrespective of MIA and sex. Notable, the levels of TNF-α were lowest in saline-treated and intermediate in fasting pigs, whereas the levels of IL-12 were the lowest in fasting pigs. The higher level of TNF-α observed in Poly(I:C)-treated relative to saline-treated pigs across MIA groups was consistent with the higher levels of serum TNF-α in four- week old pigs treated with LPS across MIA groups [3].

The serum levels of IL-18, IL-2, and IL-4 had a significant prenatal stress effect. The IL-18 concentration was lower in pigs born from PRRSV-challenged gilts relative to pigs from control gilts. This profile is in agreement with reports that the levels of IL-18 were low in the spleen and brain of MIA-exposed mice from gestation Poly(I:C) injection relative to control mice, while postnatal LPS treatment had no effect on this cytokine [60]. In addition to inflammatory indicator, serum IL-18 has been proposed as a marker for acute kidney injury [61].

Similar to the profile of IL-18, the levels of IL-2 and IL-4 were lower in pigs from PRRSV-challenged relative to control gilts (Figure 3). Consistent with our results, lower IL-2 levels were detected in the serum of one-week-old mice from Poly(I:C)-challenged females during gestation [6]. The IL-4 concentration was higher in postnatal stressed pigs from control gilts, whereas the levels were more uniform across postnatal stress groups of pigs from PRRSV-challenged gilts. This finding is in agreement with a review that noted no specific IL-4 pattern associated with MIA across studies [62], despite reports of an anti-inflammatory role of IL-4 [63,64]. 

## 5. Conclusions

The present study advances the understanding of the impact of prenatal MIA and postnatal stressors on blood biomarkers of physiological health and organ dysfunction. The experimental design enabled the characterization of combined and individual stress effects, and the identification of sex-dependent responses of serum biochemical and immune biomarkers. The identification of interacting effects offered insights into enhanced sensitivity or tolerance to stressors at 60 days of age, associated with MIA during gestation.

Pre- and postnatal stress interactions were detected for serum biochemical biomarkers of liver, muscular, and kidney function, including BUN, triglycerides, bilirubin, and phosphorous. Likewise, postnatal stressors had MIA and sex-dependent effects on the inflammatory biomarkers IFN-γ, TNF-α, IL-2, and IL-18. The detected blood biomarker patterns can guide the improvement of management practices that minimize the deleterious interaction effects of pre- and postnatal stressors. Preventive vaccination practices can reduce the risk of chronic inflammation or metabolic disorders associated with MIA alone or interacting with postnatal stressors. Similarly, feeding strategies could be adapted to ameliorate metabolic dysfunction of pigs impacted by immune activation during gestation.

## Figures and Tables

**Table 1:** *p*-values of the interaction and main effects of prenatal stress (PRRSV-challenged or control gilt), postnatal stress (Poly(I:C)-treated, saline-treated or fasting), and sex on the chemistry analyte concentrations and body weight of 60-day-old pigs.

Analyte ^1^	Prenatal Stress	Sex	Post-Natal Stress	Prenatal by Sex	Pre- by Postnatal	Postnatal by Sex	Pre- by Postnatal by Sex
AG Ratio	0.080	0.114	0.137	0.445	0.819	0.467	0.826
Albumin	0.479	0.037	0.372	0.778	0.450	0.904	0.416
ALP	0.342	0.086	0.570	0.096	0.457	0.735	0.100
Anion Gap	0.626	0.622	0.179	0.810	0.099	0.304	0.153
AST	0.234	0.917	0.028	0.656	0.322	0.090	0.298
Bicarbonate	0.288	0.261	0.010	0.926	0.297	0.718	0.659
Bilirubin	0.698	0.078	0.001	0.922	0.676	0.217	0.058
BUN	0.843	0.521	0.001	0.695	0.537	0.658	0.002
Calcium	0.282	0.153	0.005	0.985	0.427	0.959	0.001
Chloride	0.447	0.192	0.668	0.356	0.765	0.496	0.180
Cholesterol	0.632	0.117	0.005	0.751	0.810	0.503	0.008
CPK	0.820	0.004	0.023	0.922	0.676	0.217	0.058
Creatinine	0.100	0.053	0.008	0.875	0.509	0.832	0.301
GGT	0.042	0.489	0.120	0.331	0.755	0.542	0.029
GLDH	0.285	0.473	0.107	0.953	0.067	0.796	0.348
Globulin	0.430	0.581	0.098	0.694	0.630	0.305	0.667
Glucose	0.023	0.270	0.056	0.742	0.733	0.508	0.096
NaK Ratio	0.236	0.452	0.860	0.787	0.495	0.438	0.800
Phosphorus	0.566	0.121	0.005	0.741	0.480	0.969	0.001
Potassium	0.108	0.278	0.159	0.825	0.455	0.686	0.732
Protein	0.095	0.001	0.298	0.916	0.553	0.250	0.097
Sodium	0.144	0.154	0.410	0.249	0.277	0.595	0.040
Triglycerides	0.689	0.460	0.001	0.723	0.841	0.552	0.015
Body Weight	0.168	0.083	0.040	0.131	0.025	0.418	0.051

^1^ AG Ratio = albumin:globulin ratio; ALP = alkaline phosphatase; AST = aspartate amino transferase; BUN = blood urea nitrogen; CPK = creatine phosphokinase; Protein = total protein; GGT = gamma glutamyl transferase; GLDH = glutamate dehydrogenase; NaK Ratio = sodium:potassium ratio.

**Table 2:** *p*-values of the effect of postnatal stress (Poly(I:C)-treated, saline-treated or fasting) on the chemistry analyte concentrations and body weight of 60-day-old pigs, partitioned by level of prenatal stress (PRRSV-challenged or control gilt), and by sex.

Analyte ^1^	Control Female	PRRSV Female	Control Male	PRRSV Male	Control	PRRSV	Female	Male
AG Ratio	0.200	0.982	0.739	0.902	0.529	0.101	0.416	0.638
Albumin	0.692	0.560	0.698	0.536	0.077	0.052	0.871	0.425
ALP	0.427	0.945	0.122	0.226	0.692	0.010	0.860	0.251
Anion Gap	0.764	0.176	0.152	0.021	0.746	0.572	0.118	0.026
AST	0.322	0.056	0.187	0.801	0.697	0.799	0.033	0.291
Bicarbonate	0.242	0.623	0.961	0.187	0.751	0.737	0.571	0.009
Bilirubin	0.145	0.584	0.062	0.074	0.070	0.146	0.180	0.006
BUN	0.027	0.020	0.003	0.041	0.688	0.866	0.011	0.001
Calcium	0.001	0.118	0.007	0.002	0.322	0.357	0.001	0.001
Chloride	0.442	0.998	0.043	0.223	0.084	0.832	0.603	0.017
Cholesterol	0.132	0.103	0.012	0.003	0.468	0.279	0.014	0.001
CPK	0.145	0.584	0.062	0.074	0.070	0.146	0.180	0.006
Creatinine	0.179	0.297	0.097	0.783	0.317	0.440	0.442	0.151
GGT	0.075	0.839	0.099	0.009	0.803	0.249	0.284	0.008
GLDH	0.036	0.742	0.239	0.914	0.328	0.388	0.264	0.614
Globulin	0.247	0.857	0.459	0.373	0.628	0.915	0.531	0.193
Glucose	0.099	0.289	0.051	0.092	0.483	0.200	0.227	0.010
NaK Ratio	0.111	0.856	0.864	0.611	0.504	0.789	0.525	0.608
Phosphorus	0.009	0.006	0.021	0.011	0.066	0.190	0.012	0.002
Potassium	0.070	0.868	0.986	0.802	0.393	0.634	0.671	0.983
Protein	0.803	0.479	0.259	0.122	0.030	0.076	0.708	0.031
Sodium	0.034	0.939	0.032	0.226	0.042	0.815	0.030	0.004
Triglycerides	0.108	0.058	0.013	0.043	0.925	0.602	0.017	0.001
Body Weight	0.019	0.029	0.234	0.280	0.339	0.115	0.096	0.259

^1^ AG Ratio = albumin:globulin ratio; ALP = alkaline phosphatase; AST = aspartate amino transferase; BUN = blood urea nitrogen; CPK = creatine phosphokinase; GGT = gamma glutamyl transferase; GLDH = glutamate dehydrogenase; Protein = total protein; NaK Ratio = sodium:potassium ratio.

**Table 3:** *p*-values of the effect of prenatal stress (PRRSV-challenged or control gilt) on the chemistry analyte concentrations and body weight of 60-day-old pigs, partitioned the level of postnatal stress (Poly(I:C)-treated, saline-treated, or fasting), and sex.

Analyte ^1^	F ^2^ Sal	F Poly	F Fast	M Sal	M Poly	M Fast	Sal	Poly	Fast	M	F
AG Ratio	0.783	0.170	0.568	0.294	0.350	0.473	0.442	0.234	0.395	0.220	0.469
Albumin	0.706	0.300	0.659	0.500	0.390	0.823	0.530	0.274	0.926	0.466	0.654
ALP	0.785	0.198	0.127	0.083	0.381	0.680	0.703	0.426	0.180	0.594	0.081
Anion Gap	0.499	0.700	0.239	0.774	0.607	0.199	0.835	0.498	0.033	0.877	0.691
AST	0.424	0.332	0.366	0.775	0.728	0.193	0.707	0.382	0.128	0.284	0.598
Bicarbonate	0.683	0.285	0.178	0.451	0.302	0.316	0.870	0.995	0.052	0.359	0.519
Bilirubin	0.543	0.988	0.357	0.699	0.960	0.616	0.932	0.942	0.435	0.752	0.839
BUN	0.107	0.156	0.823	0.685	0.277	0.874	0.180	0.960	0.987	0.386	0.815
Calcium	0.955	0.129	0.745	0.905	0.380	0.417	0.948	0.112	0.733	0.390	0.438
Chloride	0.441	0.990	0.303	0.489	0.245	0.973	0.287	0.247	0.357	0.289	0.319
Cholesterol	0.966	0.911	0.690	0.973	0.552	0.902	0.918	0.634	0.743	0.825	0.853
CPK	0.543	0.988	0.357	0.699	0.960	0.616	0.932	0.942	0.435	0.752	0.839
Creatinine	0.642	0.136	0.239	0.176	0.735	0.770	0.255	0.594	0.539	0.523	0.732
GGT	0.079	0.549	0.909	0.270	0.066	0.080	0.098	0.379	0.208	0.032	0.434
GLDH	0.607	0.518	0.073	0.274	0.656	0.363	0.280	0.508	0.089	0.400	0.394
Globulin	0.956	0.269	0.320	0.337	0.363	0.319	0.641	0.294	0.272	0.294	0.438
Glucose	0.292	0.072	0.773	0.668	0.140	0.875	0.312	0.884	0.941	0.590	0.953
NaK Ratio	0.861	0.077	0.619	0.674	0.951	0.930	0.827	0.218	0.698	0.770	0.535
Phosphorus	0.290	0.004	0.764	0.306	0.635	0.641	0.899	0.158	0.924	0.655	0.430
Potassium	0.696	0.060	0.667	0.877	0.873	0.809	0.986	0.300	0.727	0.890	0.774
Protein	0.856	0.535	0.106	0.424	0.382	0.223	0.508	0.336	0.078	0.184	0.252
Sodium	0.201	0.072	0.798	0.760	0.037	0.785	0.088	0.097	0.898	0.263	0.049
Triglycerides	0.646	0.207	0.993	0.536	0.700	0.409	0.957	0.422	0.766	0.862	0.544
Weight	0.749	0.659	0.657	0.807	0.545	0.832	0.026	0.275	0.691	0.096	0.671

^1^ AG Ratio = albumin:globulin ratio; ALP = alkaline phosphatase; AST = aspartate amino transferase; BUN = blood urea nitrogen; CPK = creatine phosphokinase; Protein = total protein; GGT = gamma glutamyl transferase; GLDH = glutamate dehydrogenase; NaK Ratio = sodium:potassium ratio. ^2^ M = male; F = female; Sal = saline; Poly = Poly(I:C); Fast = fasting.

**Table 4:** Least square means of the chemistry analyte concentration (log transformed) and body weight of 60-day-old pigs from control gilts by postnatal stress (Poly(I:C)-treated, saline-treated, or fasting pigs) and sex group.

		Female			Male			
Analyte ^1^	Unit(log_e_)	Saline ^2^	Poly(I:C)	Fasting	Saline	Poly(I:C)	Fasting	SEM
AG Ratio		0.36	0.60	0.48	0.49	0.47	0.38	0.12
Albumin	g/dL	1.11	1.16	1.10	1.02	1.08	1.05	0.05
ALP	U/L	5.40	5.55	5.56	5.37	5.61	5.58	0.10
Anion Gap	mmol/L	2.72	2.72	2.78	2.65	2.78	2.78	0.06
AST	U/L	3.57	3.89	3.38	3.77	3.68	3.21	0.24
Bicarbonate	mmol/L	3.30	3.19	3.33	3.30	3.32	3.30	0.06
Bilirubin	mg/dL	7.78	7.97	7.11	7.49	7.23	6.40	0.40
BUN	mg/dL	1.87 ^a^	2.08 ^a^	2.52 ^a,b^	1.90 ^a^	1.78 ^a^	2.65 ^b^	0.17
Calcium	mg/dL	2.37 ^b^	2.44 ^b^	2.25 ^a^	2.34 ^a,b^	2.40 ^b^	2.24 ^a^	0.03
Chloride	mmol/L	4.77 ^a,b^	4.62 ^a^	4.81 ^b^	4.62 ^a^	4.63 ^a,b^	4.60 ^a^	0.06
Cholesterol	mg/dL	4.40 ^a,b^	4.41 ^a,b^	4.60 ^a,b^	4.29 ^a^	4.36 ^a,b^	4.63 ^b^	0.08
CPK	U/L	7.78	7.97	7.11	7.49	7.23	6.40	0.40
Creatinine	mg/dL	−0.02 ^a,b^	0.23 ^b^	0.07 ^a,b^	−0.14 ^a^	0.01 ^a,b^	0.12 ^a,b^	0.09
GGT	U/L	3.50	3.76	3.74	3.52	3.66	3.76	0.08
GLDH	U/L	−0.18	0.41	−0.42	0.01	0.47	0.04	0.28
Globulin	g/dL	0.73	0.56	0.63	0.55	0.60	0.68	0.09
Glucose	mg/dL	4.84	4.31	4.50	4.78	4.76	4.33	0.16
NaK Ratio		3.48	3.27	3.51	3.49	3.50	3.44	0.08
Phosphorus	mg/dL	2.04 ^a^	2.36 ^a,b^	2.37 ^b^	2.05 ^a^	2.02 ^a^	2.31 ^a,b^	0.08
Potassium	mmol/L	1.42 ^a^	1.68 ^b^	1.42 ^a^	1.42 ^a^	1.42 ^a^	1.44 ^a,b^	0.09
Protein	g/dL	1.64	1.61	1.61	1.52	1.56	1.59	0.04
Sodium	mmol/L	4.95	4.96	4.93	4.94	4.95	4.93	0.01
Triglycerides	mg/dL	3.82 ^a^	4.11 ^a,b^	5.05 ^a,b^	3.85 ^a^	3.79 ^a^	5.48 ^b^	0.44
Body Weight	kg	25.66	25.95	24.30	25.70	25.38	24.71	1.07

^1^ AG Ratio = albumin:globulin ratio; ALP = alkaline phosphatase; AST = aspartate amino transferase; BUN = blood urea nitrogen; CPK = creatine phosphokinase; GGT = gamma glutamyl transferase; GLDH = glutamate dehydrogenase; Protein = total protein; NaK Ratio = sodium:potassium ratio. ^2^ Least square means estimate. ^a,b^ Difference between postnatal stress and sex groups significant at *p*-value < 0.05 among pigs from control gilts.

**Table 5:** Least square means of the chemistry analyte concentration (log-transformed) and body weight of 60-day-old pigs from PRRSV-challenged gilts by postnatal stress (Poly(I:C)-treated, saline-treated, or fasting pigs) and sex group.

		Female			Male			
Analyte ^1^	Unit(log_e_)	Saline ^2^	Poly(I:C)	Fasting	Saline	Poly(I:C)	Fasting	SEM
AG Ratio		0.30	0.31	0.34	0.26	0.28	0.21	0.18
Albumin	g/dL	1.07	1.06	1.14	0.97	1.00	1.03	0.07
ALP	U/L	5.35	5.34	5.30	5.63	5.73	5.52	0.11
Anion Gap	mmol/L	2.79	2.68	2.90	2.63	2.74	2.90	0.07
AST	U/L	3.24	4.27	3.72	3.86	3.81	3.67	0.24
Bicarbonate	mmol/L	3.25	3.30	3.18	3.36	3.24	3.21	0.07
Bilirubin	mg/dL	7.34 ^a,b^	7.96 ^b^	7.61 ^b^	7.72 ^b^	7.25 ^a,b^	6.66 ^a^	0.36
BUN	mg/dL	2.32 ^a,b^	1.67 ^a^	2.58 ^b^	2.00 ^a,b^	2.05 ^a,b^	2.61 ^b^	0.19
Calcium	mg/dL	2.37	2.36	2.26	2.34	2.36	2.20	0.03
Chloride	mmol/L	4.62	4.62	4.61	4.61	4.61	4.60	0.01
Cholesterol	mg/dL	4.41 ^a^	4.39 ^a^	4.65 ^b^	4.29 ^a^	4.30 ^a^	4.64 ^b^	0.08
CPK	U/L	7.34 ^a,b^	7.96 ^b^	7.61 ^b^	7.72 ^b^	7.25 ^a,b^	6.66 ^a^	0.36
Creatinine	mg/dL	0.04	0.02	0.21	0.02	0.06	0.09	0.07
GGT	U/L	3.75 ^a,b^	3.69 ^a^	3.75 ^a,b^	3.65 ^a^	3.87 ^a,b^	3.98 ^b^	0.08
GLDH	U/L	0.06	0.16	0.33	0.44	0.32	0.41	0.23
Globulin	g/dL	0.73	0.74	0.80	0.70	0.73	0.84	0.12
Glucose	mg/dL	4.63	4.84	4.44	4.71	4.37	4.36	0.15
NaK Ratio		3.46	3.52	3.45	3.53	3.51	3.43	0.08
Phosphorus	mg/dL	2.19	1.97	2.41	1.94	2.08	2.26	0.08
Potassium	mmol/L	1.47	1.41	1.48	1.40	1.40	1.47	0.09
Protein	g/dL	1.65	1.65	1.71	1.56	1.61	1.66	0.04
Sodium	mmol/L	4.94	4.94	4.94	4.94	4.93	4.93	0.01
Triglycerides	mg/dL	4.13 ^b^	3.25 ^a^	5.04 ^c^	3.49 ^a,b^	4.01 ^a,b^	4.97 ^b,c^	0.43
Body Weight	kg	25.94	25.6	23.84	25.89	25.88	24.9	1.08

^1^ AG Ratio = albumin:globulin ratio; ALP = alkaline phosphatase; AST = aspartate amino transferase; BUN = blood urea nitrogen; CPK = creatine phosphokinase; GGT = gamma glutamyl transferase; GLDH = glutamate dehydrogenase; NaK Ratio = sodium:potassium ratio; Protein = total protein. ^2^ Least square means estimate. ^a,b,c^ Difference between postnatal stress and sex groups significant at *p*-value < 0.05 among pigs from PRRSV-challenged gilts.

**Table 6:** *p*-values of the interaction and main effects of prenatal stress (PRRSV-challenged or control gilts), postnatal stress (Poly(I:C)-treated, saline-treated, or fasting pigs), and sex on the serum cytokine concentrations (ng/mL) of 60-day-old pigs.

Cytokine	Prenatal Stress	Sex	Postnatal Stress	Prenatal × Sex	Prenatal × Postnatal	Sex × Postnatal	Pre × Postnatal × Sex
IFN-γ ^1^	0.420	0.461	0.249	0.189	0.009	0.008	0.028
IL-1α	0.190	0.140	0.226	0.361	0.158	0.559	0.481
IL-10	0.070	0.675	0.167	0.903	0.151	0.904	0.468
IL-12	0.838	0.964	0.027	0.652	0.789	0.981	0.543
IL-18	0.015	0.402	0.133	0.672	0.333	0.845	0.543
IL-1β	0.526	0.353	0.521	0.379	0.480	0.519	0.530
IL-2	0.030	0.589	0.310	0.854	0.188	0.711	0.832
IL-4	0.008	0.861	0.156	0.588	0.144	0.800	0.698
TNF-α	0.102	0.673	0.001	0.038	0.962	0.640	0.048

^1^ IFN-γ = interferon gamma; IL-1α = interleukin-1 alpha; IL-1β = interleukin 1 beta; IL-2 = interleukin 2; IL-4 = interleukin 4; IL-10 = interleukin 10; IL-12 = interleukin 12; IL-18 = interleukin 18; TNF-α = tumor necrosis factor alpha.

**Table 7:** Least square means of cytokine concentrations (ng/mL) in 60-day-old pigs from control gilts by post-stressor (Poly(I:C)-treated, saline-treated, or fasting) and sex group.

	Female			Male			
Analyte ^1^	Saline ^2^	Fasting	Poly(I:C)	Saline	Fasting	Poly(I:C)	SEM
IFN-γ	12.08	20.44	21.95	11.84	21.52	25.06	3.61
IL-1α	0.06 ^a^	0.09 ^a,b^	0.13 ^a,b^	0.08 ^a,b^	0.1 ^a,b^	0.14 ^b^	0.02
IL-10	0.25	0.45	0.87	0.45	0.51	0.7	0.20
IL-12	0.85	0.82	1.28	0.89	0.84	1.07	0.17
IL-18	0.67	0.91	1.61	0.97	1.10	1.32	0.31
IL-1β	0.15 ^a^	0.26 ^a,b^	0.50 ^b^	0.20 ^a,b^	0.32 ^a,b^	0.50 ^b^	0.10
IL-2	0.14	0.28	0.46	0.26	0.29	0.39	0.12
IL-4	0.45	1.45	1.64	0.78	1.02	1.51	0.44
TNF-α	0.37 ^a^	0.28 ^a^	1.15 ^b^	0.25 ^a^	0.34 ^a^	0.8 ^a,b^	0.14

^1^ IFN-γ = interferon gamma; IL-1α = interleukin-1 alpha; IL-1β = interleukin 1 beta; IL-2 = interleukin 2; IL-4 = interleukin 4; IL-10 = interleukin 10; IL-12 = interleukin 12; IL-18 = interleukin 18; TNF-α = tumor necrosis factor alpha. ^2^ Least square means estimate. ^a,b^ Difference between postnatal stress and sex groups significant at *p*-value < 0.05 among pigs from PRRSV-challenged gilts.

**Table 8:** Least square means of cytokine concentrations (ng/mL) of 60-day-old pigs from PRRSV-challenged gilts by post-stressor (Poly(I:C)-treated, saline-treated, or fasting), and sex group.

	Female			Male			
Analyte ^1^	Saline ^2^	Fasting	Poly(I:C)	Saline	Fasting	Poly(I:C)	SEM
IFN-γ	29.17 ^b^	9.97 ^a^	14.32 ^a,b^	7.25 ^a^	16.3 ^a,b^	15.94 ^a,b^	5.06
IL-1α	0.05	0.05	0.04	0.06	0.16	0.07	0.04
IL-10	0.4	0.24	0.27	0.33	0.34	0.41	0.15
IL-12	0.96	0.8	0.99	0.89	0.82	1.15	0.17
IL-18	0.5	0.51	0.5	0.63	0.68	0.82	0.26
IL-1β	0.12	0.16	0.12	0.19	0.50	0.29	0.10
IL-2	0.12	0.16	0.11	0.19	0.17	0.16	0.08
IL-4	0.4	0.38	0.36	0.51	0.58	0.51	0.27
TNF-α	0.17 ^a^	0.13 ^a^	0.53 ^a,b^	0.15 ^a^	0.23 ^a^	1.07 ^b^	0.15

^1^ IFN-γ = interferon gamma; IL-1α = interleukin-1 alpha; IL-1β = interleukin 1 beta; IL-2 = interleukin 2; IL-4 = interleukin 4; IL-10 = interleukin 10; IL-12 = interleukin 12; IL-18 = interleukin 18; TNF-α = tumor necrosis factor alpha. ^2^ Least square means estimate. ^a,b^ Difference between postnatal stress and sex groups significant at *p*-value < 0.05 among pigs from PRRSV-challenged gilts.

## Data Availability

Data is available upon request from the corresponding author.

## Supplementary Materials

The following are available online at https://www.mdpi.com/article/10.3390/ani11040987/s1, Table S1: Nutrient composition of the diets. Table S2: Descriptive statistics of the serum chemistry (natural log-transformed) and cytokine biomarkers analyzed for all samples and for baseline pigs.
