# Supplementary material for: Biochemistry and Immune Biomarkers Indicate Interacting Effects of Pre- and Postnatal Stressors in Pigs across Sexes
Source: Animals (Basel). 2021 Apr 1;11(4):987. doi: 10.3390/ani11040987 (PMC8067328; doi:10.3390/ani11040987)
Supplement: Supplementary file 1 [file animals-11-00987-s001.zip › rymutchemcytopd60_animalsSuppTablesfeb2421docx.docx]

Table S1. Nutrient composition of the diets.

| Ingredients^1^ % | Gestation | Lactation | Postweaning day 22-30 | Postweaning day 31-40 | Postweaning day 41-59 | |
| --- | --- | --- | --- | --- | --- | --- |
| Corn | 77.15 | 67.40 | 30.74 | 46.94 | 57.47 |  |
| Soybean meal, 48 % CP | 11.55 | 24.13 | 20.39 | 23.87 | 31.49 |  |
| Beet pulp | 7.00 | 0.00 | 0.00 | 0.00 | 0.00 |  |
| Blood plasma | 0.00 | 0.00 | 7.56 | 3.02 | 0.00 |  |
| Whey, dried | 0.00 | 0.00 | 25.19 | 20.15 | 5.05 |  |
| Milk, Lactose | 0.00 | 0.00 | 10.08 | 0.00 | 0.00 |  |
| Choice white grease | 1.00 | 5.01 | 3.02 | 3.02 | 3.03 |  |
| Limestone | 0.75 | 0.89 | 1.15 | 1.10 | 0.91 |  |
| Dicalcium phosphate | 1.90 | 1.86 | 0.63 | 0.86 | 1.36 |  |
| Lysine HCL | 0.00 | 0.05 | 0.04 | 0.05 | 0.02 |  |
| DL-Met | 0.00 | 0.00 | 0.14 | 0.07 | 0.02 |  |
| Salt | 0.00 | 0.00 | 0.10 | 0.10 | 0.00 |  |
| Cu sulfate | 0.00 | 0.00 | 0.00 | 0.00 | 0.08 |  |
| ZnO | 0.00 | 0.00 | 0.40 | 0.26 | 0.00 |  |
| Swine trace minerals premix^2^ | 0.35 | 0.35 | 0.35 | 0.35 | 0.35 |  |
| Vitamin premix^3^ | 0.20 | 0.20 | 0.20 | 0.20 | 0.20 |  |
| Sow pac^4^ | 0.10 | 0.10 | 0.00 | 0.00 | 0.00 |  |
| Total | 100.00 | 100.00 | 100.00 | 100.00 | 100.00 |  |

^1^ Diets formulated to meet or exceed the NRC 2012 requirements for swine.

^2^ Ingredients: copper, iodine, iron, manganese, selenium, zinc.

^3^ Ingredients: vitamin A, vitamin D, vitamin E, vitamin K, niacin, panothenic acid, riboflavin, and vitamin B12

^4^ Ingredients: vitamin A, choline, D-biotin, and folic acid.

Table S2. Descriptive statistics of the serum chemistry (natural log-transformed) and cytokine biomarkers analyzed for all samples and for baseline pigs.

|  | Unit | All Samples |  | Baseline^1^ Samples |  |
| --- | --- | --- | --- | --- | --- |
| Variable^2^ |  | Mean | SE | Mean | SE |
| AGRatio |  | 0.432 | 0.027 | 0.380 | 0.056 |
| Albumin | g/dl | 1.090 | 0.010 | 1.071 | 0.024 |
| ALP | U/l | 5.519 | 0.021 | 5.443 | 0.049 |
| AnionGap | mmol/l | 2.748 | 0.011 | 2.661 | 0.029 |
| AST | U/l | 3.623 | 0.044 | 3.559 | 0.103 |
| Bicarbonate | mmol/l | 3.289 | 0.009 | 3.348 | 0.022 |
| Bilirubin | mg/dl | -1.523 | 0.059 | -1.949 | 0.111 |
| BUN | mg/dl | 2.109 | 0.032 | 1.907 | 0.064 |
| Calcium | mg/dl | 2.339 | 0.007 | 2.360 | 0.009 |
| Chloride | mmol/l | 4.645 | 0.016 | 4.653 | 0.035 |
| Cholesterol | mg/dl | 4.451 | 0.015 | 4.342 | 0.032 |
| CPK | U/l | 7.345 | 0.070 | 7.533 | 0.182 |
| Creatinine | mg/dl | 0.047 | 0.012 | -0.014 | 0.025 |
| GGT | U/l | 3.711 | 0.015 | 3.598 | 0.027 |
| GLDH | U/l | 0.159 | 0.041 | 0.072 | 0.090 |
| Globulin | g/dl | 0.658 | 0.020 | 0.688 | 0.043 |
| Glucose | mg/dl | 4.622 | 0.022 | 4.758 | 0.035 |
| NaKRatio |  | 3.503 | 0.008 | 3.509 | 0.012 |
| Phosphorous | mg/dl | 2.151 | 0.014 | 2.054 | 0.025 |
| Potassium | mmol/l | 1.425 | 0.009 | 1.424 | 0.011 |
| Protein | g/dl | 1.612 | 0.007 | 1.605 | 0.017 |
| Sodium | mmol/l | 4.940 | 0.001 | 4.945 | 0.003 |
| Triglycerides | mg/dl | 4.189 | 0.073 | 3.562 | 0.126 |
| Body Weight | kg | 25.274 | 0.434 | 25.652 | 1.210 |
| IFNγ | ng/ml | 46.918 | 2.231 | 59.735 | 12.237 |
| IL-1α | ng/ml | 0.181 | 0.009 | 0.140 | 0.022 |
| Il-1β | ng/ml | 0.635 | 0.043 | 0.444 | 0.066 |
| IL-2 | ng/ml | 0.916 | 0.063 | 0.640 | 0.141 |
| IL-4 | ng/ml | 3.064 | 0.231 | 1.674 | 0.351 |
| IL-10 | ng/ml | 1.442 | 0.090 | 0.956 | 0.181 |
| IL-12 | ng/ml | 0.608 | 0.013 | 0.584 | 0.034 |
| IL-18 | ng/ml | 2.224 | 0.160 | 1.966 | 0.299 |
| TNFα | ng/ml | 0.365 | 0.018 | 0.313 | 0.058 |

^1^Baseline includes saline-treated pigs from control gilts.

^2^AG Ratio = Albumin Globulin Ratio; ALP = Alkaline Phosphatase ; AST = aspartate amino transferase; BUN = blood urea nitrogen; CPK = creatine- phosphokinase; GGT = gamma glutamyl transferase; GLDH = glutamate dehydrogenase; NaK Ratio = Sodium Potassium Ratio; Protein = total protein; IFNγ = interferon gamma; IL-1α = interleukin-1 alpha; IL-1β = interleukin 1 beta; IL-2 = interleukin 2; IL-4 = interleukin 4; IL-10 = interleukin 10; IL-12 = interleukin 12; IL-18 = interleukin 18; TNFα = tumor necrosis factor alpha; chemistry analytes were analyzed using the natural logarithmic transformation.
